# Sex-Specific Systemic Signatures in Parkinson’s Disease: Integrated Biochemical and Metabolomic Evidence

**DOI:** 10.3390/biomedicines14071511

**Published:** 2026-07-04

**Authors:** Alessandro Pistone, Martina Rosa, Maria Antonietta Castiglione Morelli, Licia Viggiani, Angelo Antonini, Luigi Bubacco, Faustino Bisaccia, Angela Ostuni

**Affiliations:** 1 Department of Basic and Applied Sciences, University of Basilicata, 85100 Potenza, Italy; alessandro.pistone@unibas.it (A.P.); martina.rosa@unibas.it (M.R.); maria.castiglione@unibas.it (M.A.C.M.); licia.viggiani@unibas.it (L.V.); 2 Neurodegenerative Disease Unit, Centre for Rare Neurological Diseases (ERN-RND), Department of Neuroscience, Padua Neuroscience Center (PNC), University of Padova, 35128 Padova, Italy; angelo.antonini@unipd.it; 3 IRCCS, San Camillo Hospital, 30126 Venice, Italy; 4 Department of Biology, University of Padova, 35128 Padova, Italy; luigi.bubacco@unipd.it; 5 Department of Health Sciences, University of Basilicata, 85100 Potenza, Italy; faustino.bisaccia@unibas.it

**Keywords:** Parkinson’s disease, sex differences, blood-based biomarkers, systemic biomarkers, oxidative stress, inflammation, α-synuclein, metabolomics, precision medicine

## Abstract

**Background/Objectives**: Parkinson’s disease (PD) exhibits marked sexual dimorphism, with a higher incidence and earlier onset in men than in women. However, the impact of biological sex on systemic molecular alterations in PD remains poorly understood. This pilot study aimed to identify sex-specific circulating signatures associated with PD. **Methods**: Serum samples from a selected cohort of PD patients and healthy controls (HC) of both sexes were analyzed using an integrated biochemical and ^1^H NMR-based metabolomic approach. Oxidative stress markers, antioxidant proteins, inflammatory mediators, matrix metalloproteinases, α-synuclein species, and circulating antibodies were evaluated. **Results**: This analysis indicated that, while global oxidative stress markers were unchanged, sex-related differences in antioxidant pathways were observed as suggested by the reduced Nrf2 expression observed in PD females and increased IL-6 levels, above all in male PD patients. MMP3 levels were significantly higher in female PD patients compared with males. Male patients showed higher levels of 52 kDa protease-resistant α-synuclein species, while females exhibited increased antibody titers against both monomeric and aggregated forms. Metabolomic profiling suggested a disease-associated metabolic remodeling in PD, with distinct sex-related metabolic signatures and a more pronounced and widespread metabolic dysregulation in males. **Conclusions**: These findings suggest that biological sex may contribute to systemic molecular heterogeneity in PD, with trends indicating more pronounced inflammatory and metabolic alterations in males and distinct immune-related responses in females. Given the exploratory nature of the study and the limited sample size, these observations should be interpreted cautiously and require validation in larger, independent cohorts. Nevertheless, the results support the importance of considering sex-related molecular differences in future biomarker studies and precision medicine approaches for PD.

## 1. Introduction

Parkinson’s disease (PD) is the second most prevalent neurodegenerative disorder worldwide, after Alzheimer’s disease, representing a growing global health burden. It is characterized by the progressive loss of dopaminergic neurons in the substantia nigra pars compacta and the accumulation of pathological α-synuclein, whose oligomeric and fibrillary forms disrupt multiple cellular processes, including mitochondrial function, vesicular trafficking, and inflammatory responses.

Although the etiology is multifactorial, involving genetic, environmental, immunological, and metabolic components, increasing attention has been directed towards the identification of reliable biomarkers for early diagnosis, patient stratification, and disease monitoring [1,2,3]. In this context, blood-based biomarkers represent a promising and minimally invasive alternative to cerebrospinal fluid analysis, despite current limitations in sensitivity and specificity [4,5].

Recent research has highlighted biological sex as a critical determinant of disease risk and progression. Epidemiological evidence consistently reports that the prevalence of PD is approximately 1.5 times higher in men than in women, with an age-adjusted incidence nearly twice as high in men [3,6]. Clinical manifestations also differ: women typically present a later onset of symptoms, often attributed to the neuroprotective effects of estrogens on dopaminergic neurons, mitochondrial function, and oxidative stress responses [7,8,9].

Beyond hormonal influences, intrinsic biological factors such as sex chromosome composition (XX vs. XY) and sexually dimorphic gene expression significantly contribute to differential vulnerability. Genes overexpressed in females are mainly associated with signal transduction and protection against oxidative stress, whereas those overexpressed in males are more frequently linked to neurodegenerative pathways [10]. Additionally, sex-specific differences have been observed in immune responses, with male patients often exhibiting a more pronounced pro-inflammatory profile and altered cytokine levels [11,12].

Despite compelling evidence of sex-related differences in PD, the integration of sex as a biological variable in research remains limited, with a historical predominance of male-based models contributing to an incomplete understanding of female-specific mechanisms and therapeutic responses [13].

Circulating biomarkers provide a valuable opportunity to characterize systemic molecular alterations that may reflect central nervous system pathology. In this context, identifying sex-specific signatures in peripheral fluids is essential for improving diagnostic precision and advancing personalized approaches within gender medicine. However, sex-stratified systemic studies in PD remain scarce, particularly those integrating multiple molecular layers. To address this gap, we designed an exploratory pilot study to identify sex-dependent molecular signatures in PD.

Using an integrated biochemical and metabolomic approach, we analyzed oxidative stress markers, inflammatory mediators, and metabolic pathways known to be dysregulated in PD [4,9], with the aim of elucidating how biological sex shapes systemic responses to α-synuclein pathology and contributes to disease heterogeneity.

## 2. Materials and Methods

### 2.1. Serum Sample Collection

Sera were obtained from the Padova Biobank, Department of Biomedical Sciences, University of Padova. All sera were kept frozen at –80 °C until analysis. The study was approved by the local Ethics Committee (Comitato Etico Territoriale Area Centro Est Veneto; protocol code 6045/AO/24) and authorized by the Azienda Ospedale-Università Padova, Italy (Deliberation No. 2134, 25 October 2024). Written informed consent was obtained from all participants prior to inclusion in the study and in accordance with the Declaration of Helsinki.

Human blood serum samples were obtained from 9 healthy controls (HC) without neurological diagnosis (5 males and 4 females; age 63.8 ± 5.5 and 54.5 ± 9.4 years, respectively) and from Parkinson’s disease (PD) patients (17 males and 5 females; age 59.7 ± 8.8 and 63.0 ± 4.6 years at sample collection, respectively). Years since symptom onset were summarized using median and range: in PD males the median duration was 5 years (range 0–15), while in PD females it was 7 years (range 1–22).

### 2.2. Evaluation of Oxidative Balance

Systemic oxidative balance was assessed using the D-ROMs and BAP tests (Diacron Diagnostic International Srl, Grosseto, Italy) to measure reactive oxygen metabolites and biological antioxidant potential, respectively. All assays were performed according to the manufacturer’s protocols.

### 2.3. Western Blot Analysis

To reduce albumin interference, serum samples were subjected to depletion according to [14]. Briefly, after cold acetone/TCA precipitation, samples were incubated at −20 °C, centrifuged and washed with acetone. Pellets were resuspended in PBS and protein concentration was determined by Bradford assay. Equal amounts of protein (8 µg) were separated by SDS-PAGE and transferred onto 0.2 µm nitrocellulose membranes using a Trans-Blot Turbo system (Bio-Rad, Hercules, CA, USA). Membranes were stained with Ponceau S (Sigma-Aldrich, St. Louis, MO, USA) to verify transfer efficiency. After blocking with 5% non-fat milk in PBS with 0.05% Tween 20 (PBST), membranes were incubated overnight at 4 °C with primary antibodies: 1:1000 anti-α-tubulin monoclonal antibody (cat no. 11H10, Cell Signaling Technology, Inc., Danvers, MA, USA); 1:5000 anti-Nrf2 polyclonal antibody (cat no. 16396-1-AP, Proteintech, Manchester, UK); 1:800 anti-UCP2 polyclonal antibody (cat no. 11081-1-AP, Proteintech, Manchester, UK); 1:100 anti-NQO1 (cat no. SC-32773, Santa Cruz Biotechnology, Inc., Dallas, TX, USA); 1:20,000 anti-SOD2 polyclonal antibody (cat no. 66474-1-Ig, Proteintech, Manchester, UK); 1:1000 anti-α-synuclein polyclonal antibody (cat no. PA1-18264, Invitrogen, Carlsbad, CA, USA); 1:1000 anti-MMP3 polyclonal antibody (cat no. 17873-1-AP, Proteintech, Manchester, UK). After washing, membranes were incubated with HRP-conjugated secondary antibodies.

Signals were detected using chemiluminescence reagents ECL™ Western Blotting Detection Reagents (GE Healthcare, Chicago, IL, USA) or SuperSignal™ West Pico PLUS Chemiluminescent Substrate (Thermo Scientific, Waltham, MA, USA) and acquired with a ChemiDoc™ XRS system (Bio-Rad, Hercules, CA, USA). Densitometric analysis was performed using ImageJ (version 1.53m; National Institutes of Health, Bethesda, MD, USA (α-tubulin was used as an internal reference for densitometric normalization based on preliminary experiments, demonstrating a detectable signal across all analyzed serum samples following albumin depletion. For each sample, densitometric values were normalized to the corresponding α-tubulin signal obtained from the same membrane. All Western blot experiments were performed using the same protocol, antibody dilutions and detection procedures.

### 2.4. Measurement of IL-6

Serum IL-6 levels were measured using a commercial human IL-6 ELISA kit (Proteintech, 6 Atherton Street, M3 3GS, Manchester, UK) following the manufacturer’s instructions.

### 2.5. Gelatin Zymography for MMP9 Activity

MMP9 enzymatic activity was assessed by gelatin zymography as previously reported [15]. Total protein concentration in serum samples was determined by Bradford assay. A total of 10 µg of protein was mixed with non-reducing sample buffer (50 mM Tris-HCl pH 6.8, 10% glycerol, 4% SDS, 0.002% bromophenol blue) and separated on 8% SDS-polyacrylamide gels containing 0.2% gelatin from bovine skin (Sigma-Aldrich, St. Louis, MO, USA). Electrophoresis was performed at 150 V at 4 °C. Gels were washed twice in 2.5% Triton X-100 to remove SDS and incubated overnight at 37 °C in activation buffer (50 mM Tris-HCl pH 6.8, 10 mM CaCl_2_, 1% Triton X-100).

Gels were stained with 0.25% Coomassie Brilliant Blue R-250 and 0.05% Coomassie Brilliant Blue G-250 (Sigma-Aldrich, St. Louis, MO, USA). Gelatinolytic activity was detected as clear bands against the stained background. Images were acquired and presented in grayscale.

### 2.6. Proteinase K Digestion of α-Synuclein

Serum samples were diluted 1:2 in PBS and protein concentration was determined by Bradford assay. A total of 20 µg of protein was incubated with Proteinase K (final concentration 10 µg/mL; Sigma-Aldrich, St. Louis, MO, USA) at 37 °C for 20 min [16]. The reaction was stopped by heating at 95 °C for 5 min. Samples were centrifuged at 12,000 × g for 3 min, and pellets were mixed with Laemmli buffer (60 mM Tris-HCl pH 6.8, 10% glycerol, 2% SDS, 1% dithiothreitol (DTT) and 0.002% bromophenol blue) and subjected to SDS-PAGE.

Digestion patterns were analyzed by Western blot. Analysis of immunoreactive bands was conducted by ImageJ (version 1.53m; National Institutes of Health, Bethesda, MD, USA)and normalized for total protein. Undigested sera were used as controls.

### 2.7. Preparation of α-Synuclein Species

Recombinant human α-synuclein was prepared in two aggregated and fibrillar forms. Aggregated α-synuclein was prepared as previously described [17]. Briefly, a 0.7 mM α-synuclein solution was subjected to constant stirring (200 rpm) at 45 °C for 2 h. Fibrillar alpha-syn (early-stage fibrils) was prepared according to Chen et al. [18] with some modifications; monomeric α-synuclein (100 μM) was reconstituted in 1× PBS (pH 7.4) supplemented with 0.5 M ammonium sulfate and 10% (*v*/*v*) 2-methyl-2,4-pentanediol (MPD) (Sigma-Aldrich, St. Louis, MO, USA). The solution was incubated at 37 °C for 3 days under constant agitation (200 rpm). Fibrils were isolated by centrifugation at 10,000 rpm for 15 min, washed once, and resuspended in PBS.

All aliquots were stored at −80 °C. Before experimental use, aliquots were thawed on ice for 30 min and equilibrated at room temperature for an additional 30 min.

### 2.8. Evaluation of Anti-α-Synuclein Antibodies

Antibodies against different α-synuclein conformers (monomers, oligomers, and fibrils) were quantified by in-house indirect enzyme-linked immunosorbent assay (ELISA).

Polystyrene microplates (Costar Corning, NY 14831, USA) were pre-treated with 5% (*v*/*v*) glutaraldehyde (Sigma-Aldrich, St. Louis, MO, USA) for 1 h at room temperature and washed five times with PBS. A total of 2 µg of α-synuclein species was immobilized in each well and incubated overnight at 4 °C. After washing, nonspecific binding sites were blocked with 5% skimmed milk in PBS for 2 h at room temperature. Serum samples were diluted 1:50 in blocking buffer and incubated for 1 h at room temperature. After washing with PBS containing 0.05% Tween-20 (PBST), plates were incubated with HRP-conjugated anti-human IgG secondary antibody (Sigma, A8792; 1:5000) for 1 h at room temperature. After additional washes, 3,3′,5,5′-tetramethylbenzidine (TMB) (Sigma-Aldrich, St. Louis, MO, USA) substrate was added and the reaction was stopped with 1 M sulfuric acid. Absorbance was measured at 450 nm using a Multiskan™ GO microplate reader (Thermo Scientific, Waltham, MA, USA). Background signals from wells without antigen were subtracted from each measurement.

### 2.9. NMR-Based Metabolomic Analysis

Serum samples were thawed on ice prior to analysis. A total of 20 µL of serum was mixed with 580 µL of D_2_O and 5 µL of TSP (3-trimethylsilyl propionic acid-d4 sodium salt), used as both a chemical shift reference (δ = 0 ppm) and an internal standard for quantitative analysis. Samples were transferred to 5 mm NMR tubes (ST500, NORELL, Inc., Morganton, NC, USA).

^1^H NMR spectra were acquired at 25 °C using a Varian Inova 500 MHz spectrometer (Varian, Palo Alto, CA, USA). Spectra were collected with 100 scans, 16K data points, a spectral width of 5995 Hz, and a relaxation delay of 5 s.

Spectra were processed using NMR Suite 8.6 (Chenomx Inc., Edmonton, Alberta, Canada). Data were Fourier transformed (32K points, 1 Hz line broadening), phase-corrected, and baseline-corrected. Metabolite identification was performed using the Profiler module of Chenomx and supported by literature data. The water region (4.7–5.1 ppm) and TSP signal were excluded from the analysis.

### 2.10. Multivariate Analysis

Multivariate analysis was performed as reported above [19]. NMR data were imported into the program SIMPCA-P+ (version 12; Umetrics AB, Umeå, Sweden)) and subjected to pre-treatment with Pareto scaling (/√SD). Preliminarily, an unsupervised Principal Component Analysis (PCA) model was built on the entire data set. A supervised model was then built with latent structure-discrimination analysis (PLS-DA) using the two different groups HC and PD, subdivided by sex. The overall quality of the model obtained by PLS-DA was evaluated by the R2 and Q2 values, where R2 measures the goodness of fit and displays the explained variation by components and Q2 gives an indication of the goodness of predicted model. The PLS-DA model was validated using permutation test to assess the risk of model overfitting. Models generated after random permutation of the y-variables showed lower R^2^ and Q^2^ values than the original model, indicating lower predictive performance and supporting the robustness of the classification model (Supplementary Figure S1).

The heatmap reported in Figure 9 was calculated with Morpheus software (https://software.broadinstitute.org/morpheus, accessed on 30 March 2026).

### 2.11. Statistical Analysis

Given the limited sample size, non-parametric statistical analyses were performed. Data are presented as box-and-whisker plots, where the center line represents the median, the box indicates the interquartile range, the lower and upper box boundaries correspond to the first and third quartiles, respectively, and the whiskers represent the minimum and maximum values. Individual data points are shown. Statistical significance was assessed using the Wilcoxon-Mann-Whitney test. Statistical significance was set at *p* < 0.05. Given the exploratory nature of this pilot study, no formal correction for multiple comparisons was applied. All statistical analyses were performed using GraphPad Prism version 8.0 (GraphPad Software, San Diego, CA, USA).

## 3. Results

### 3.1. Oxidative Stress and Inflammatory Biomarkers in Serum

**Figure 1:**
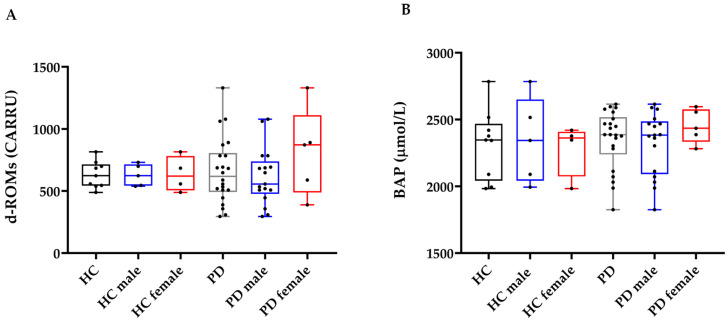
Evaluation of oxidative stress levels in serum samples from healthy controls (HC) and Parkinson’s disease (PD) patients. (**A**) Measure of hydroperoxide level by D-ROMs assay; UCARR indicates the “Carratelli Unit”, an arbitrary unit, and 1 U CARR corresponds to the color development caused by a H_2_O_2_ solution at a concentration of 0.08%; (**B**) measure of the antioxidant power by BAP test. Data are presented as box-and-whisker plots showing median, interquartile range, minimum and maximum values, together with individual data points. Statistical analysis was performed using the Wilcoxon-Mann-Whitney test. No significant differences were detected between groups.

To investigate whether systemic redox balance is altered in PD and modulated by biological sex, oxidative stress and antioxidant capacity were first assessed. The D-ROMs test, which measures circulating reactive oxygen metabolites, revealed no significant differences between PD and HC, either in the overall cohort or after stratification by sex (Figure 1A). Similarly, the biological antioxidant potential (BAP) assay showed no significant variation between groups, indicating that the global serum oxidative status is not markedly altered in PD at the systemic level (Figure 1B).

To further assess molecular alterations, some key regulators of the antioxidant response were analyzed. Nrf2 controls the expression of antioxidant and cytoprotective genes, including NQO1 and SOD2, which are involved in oxidative stress responses and the modulation of inflammation [20].

**Figure 2:**
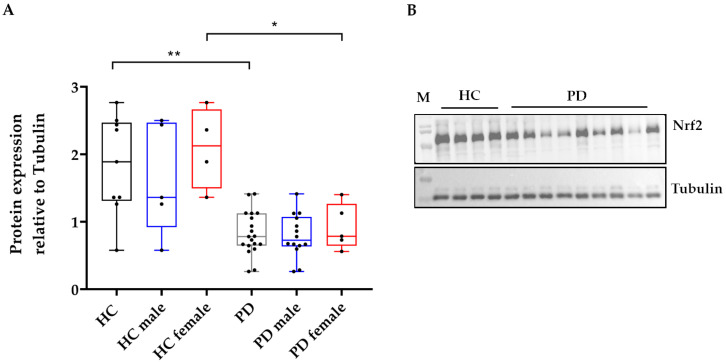
Nrf2 expression levels in serum samples from healthy controls (HC) and Parkinson’s disease (PD) patients. (**A**) Densitometric analysis of Western blot bands, normalized to α-tubulin, in HC and PD subjects, stratified by sex (male and female). Data are presented as box-and-whisker plots showing the median, interquartile range, minimum and maximum values, together with individual data points. (**B**) Representative Western blot showing Nrf2 and α-tubulin protein levels. Statistical analysis was performed using the Wilcoxon-Mann-Whitney test. Significant differences are indicated as * *p* < 0.05 and ** *p* < 0.01.

In PD patients, a decreasing trend in Nrf2 expression was detected, reaching statistical significance in PD females compared with HC females (Figure 2).

**Figure 3:**
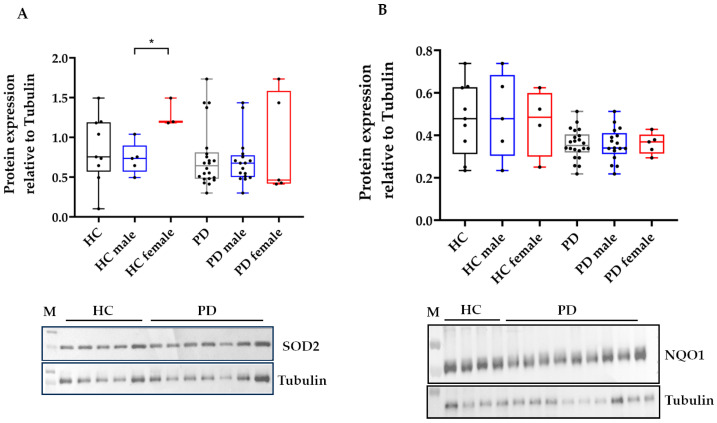
Expression levels in serum samples from healthy controls (HC) and Parkinson’s disease (PD) patients of (**A**) SOD2 and (**B**) NQO1. Densitometric analysis of Western blot bands, normalized to α-tubulin, in HC and PD subjects, stratified by sex (male and female). Data are presented as box-and-whisker plots showing median, interquartile range, minimum and maximum values, together with individual data points. Representative Western blot showing protein levels. Statistical analysis was performed using the Wilcoxon-Mann-Whitney test. Significant differences are indicated as * *p* < 0.05.

Moreover, HC females exhibited higher SOD2 expression levels than their male counterparts; however, in PD patients, SOD2 expression did not differ between PD subgroups or in comparison with HCs (Figure 3A). In PD patients, regardless of sex, a non-significant downward trend in NQO1 expression was observed (Figure 3B).

**Figure 4:**
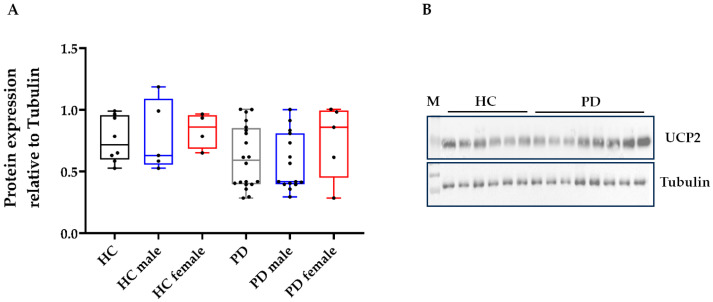
UCP2 expression levels in serum samples from healthy controls (HC) and Parkinson’s disease (PD) patients. (**A**) Densitometric analysis of Western blot bands, normalized to α-tubulin, in HC and PD subjects, stratified by sex (male and female). Data are presented as box-and-whisker plots showing the median, interquartile range, minimum and maximum values, together with individual data points. (**B**) Representative Western blot showing UCP2 and α-tubulin protein levels. Statistical analysis was performed using the Wilcoxon-Mann-Whitney test. No significant differences were detected between groups.

The mitochondrial protein UCP2, which contributes to the regulation of mitochondrial membrane potential and reactive oxygen species (ROS) production [21], showed no significant variation between groups (Figure 4).

**Figure 5:**
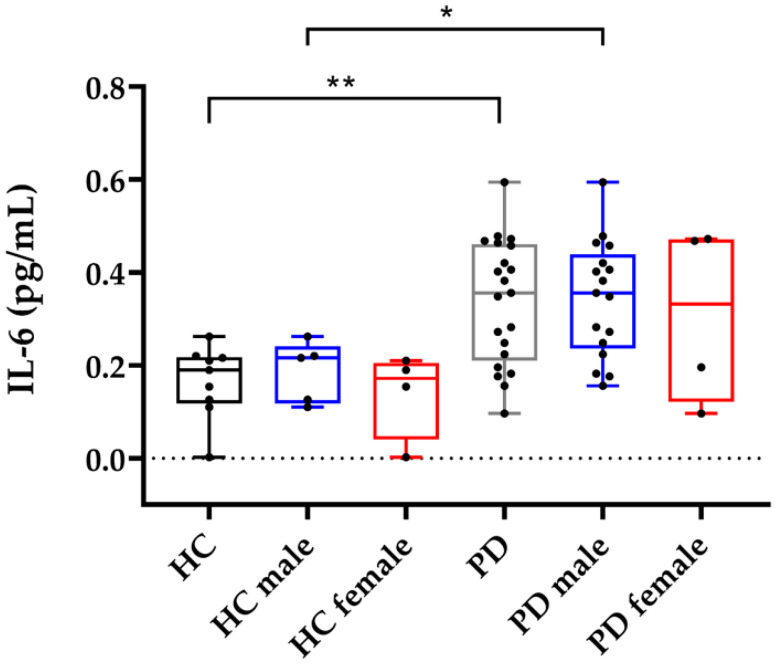
IL-6 concentrations in serum samples from healthy controls (HC) and Parkinson’s disease (PD) patients. Concentrations were measured by ELISA sandwich assay. Data are presented as box-and-whisker plots showing median, interquartile range, minimum and maximum values, together with individual data points. Statistical analysis was performed by Wilcoxon-Mann-Whitney test. * *p* < 0.05. ** *p* < 0.01.

Inflammatory status was evaluated by measuring serum interleukin-6 (IL-6) levels [22] using ELISA. IL-6 concentrations were significantly elevated in PD patients compared with HC, with the most pronounced increase observed in male PD patients (Figure 5).

**Figure 6:**
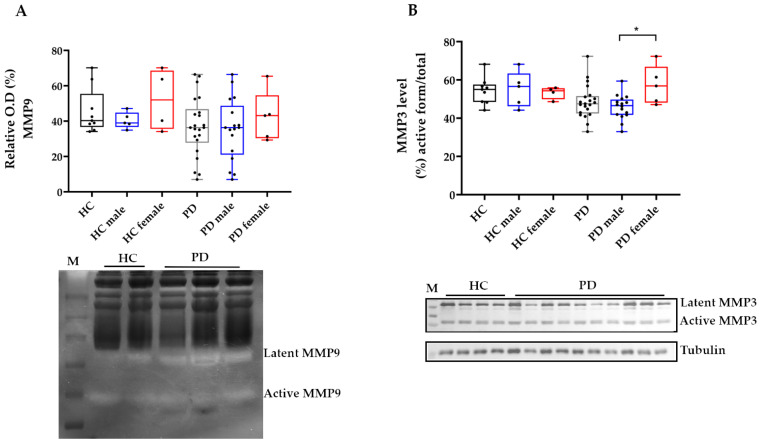
Matrix metalloproteinases in serum samples from healthy controls (HC) and Parkinson’s disease (PD) patients. (**A**) MMP9 activity was evaluated by gelatin zymography. Densitometric analysis of the bands is expressed as optical density (OD) of the active form normalized to total activity. A representative zymogram is shown. (**B**) MMP3 expression was analyzed by Western blot. Densitometric analysis of immunoreactive bands was normalized to α-tubulin and expressed as a percentage of the active form relative to total forms. A representative blot is shown. Data are presented as box-and-whisker plots showing median, interquartile range, minimum and maximum values, together with individual data points. Statistical analysis was performed using the Wilcoxon-Mann-Whitney test. * *p* < 0.05.

Given the established role of matrix metalloproteinases (MMPs) in blood-brain barrier disruption, serum levels of MMP9 and MMP3 activity were analyzed [23]. Gelatin zymography showed no significant differences in MMP9 activity between PD and HC groups, both regardless of sex and when segregated by sex (Figure 6A). In contrast, levels of active MMP3 were significantly increased in female PD patients compared with their male counterparts (Figure 6B).

### 3.2. Protease-Resistant α-Synuclein Species and Humoral Immune Response in Serum

Given the structural heterogeneity of α-synuclein and its potential impact on both antigen stability and immune recognition, we investigated the presence of protease-resistant α-synuclein species and the corresponding humoral response against distinct α-synuclein conformers in serum from Parkinson’s disease patients and controls.

**Figure 7:**
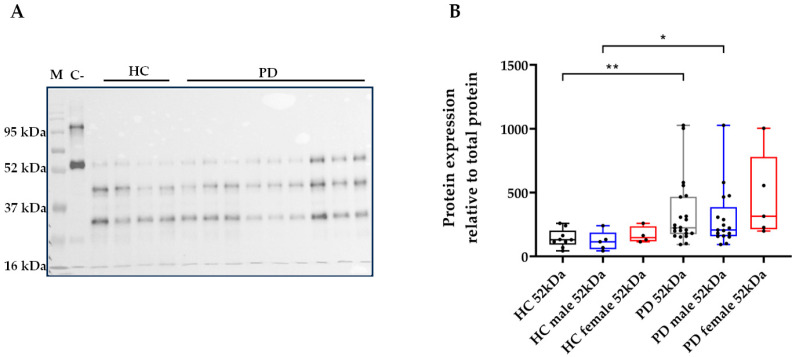
α-Synuclein profile in serum samples from healthy controls (HC) and Parkinson’s disease (PD) patients. (**A**) Representative Western blot of α-synuclein following controlled Proteinase K digestion. A negative control (C−), consisting of serum not subjected to proteolytic treatment, was included. (**B**) Densitometric analysis of the immunoreactive band at 52 kDa in HC and PD subjects, analyzed regardless of sex and stratified by sex. Protein levels were normalized to total protein. Data are presented as box-and-whisker plots showing median, interquartile range, minimum and maximum values, together with individual data points. Statistical analysis was performed using the Wilcoxon-Mann-Whitney test. Significant differences are indicated as * *p* < 0.05 and ** *p* < 0.01.

To assess disease-associated, structurally constrained α-synuclein species, serum samples were subjected to controlled proteinase K digestion followed by Western blot analysis (Figure 7A). PD sera displayed a distinct immunoreactive profile characterized by multiple protease-resistant α-synuclein species. Among these, a band at approximately 52 kDa showed the most evident variation between PD patients and HC subjects and was therefore selected for further quantitative analysis. Quantification revealed significantly higher levels of the 52 kDa species in male PD patients compared to HC of the same sex, suggesting a sex-dependent difference in the abundance of protease-resistant α-synuclein species (Figure 7B). Data for the remaining bands are provided in Supplementary Figure S2.

**Figure 8:**
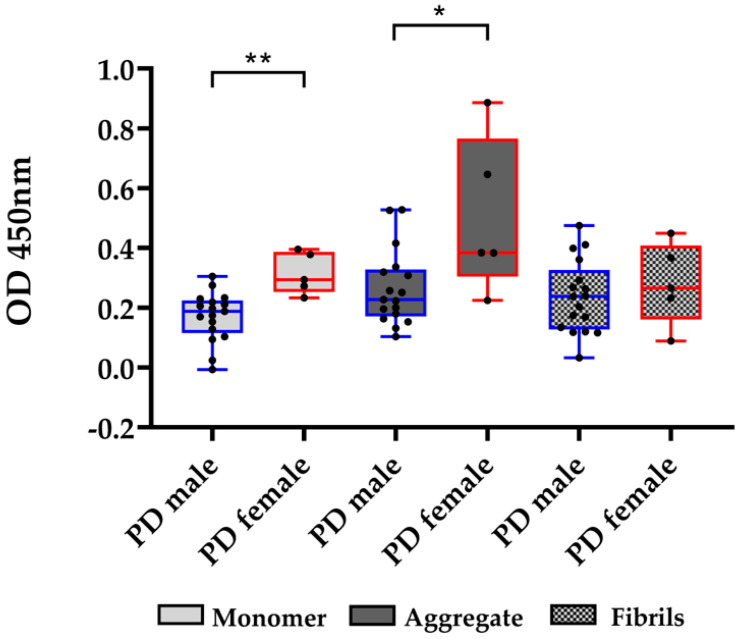
α-Synuclein antibodies in serum samples from healthy controls (HC) and Parkinson’s disease (PD) patients. The presence of circulating antibodies against different forms of α-synuclein was measured by an in-house indirect ELISA. Data are presented as box-and-whisker plots showing median, interquartile range, minimum and maximum values, together with individual data points. Statistical analysis was performed using the Wilcoxon-Mann-Whitney test. Comparisons between PD patients of opposite sexes are indicated (* *p* < 0.05; ** *p* < 0.01).

To determine whether these structurally constrained species are mirrored by systemic immune responses, we next assessed circulating antibodies against different conformational states of α-synuclein using an in-house indirect ELISA with recombinant monomeric, oligomeric, and fibrillar forms of the protein as antigens. The complete dataset is provided in Supplementary Figure S3; only statistically significant comparisons are reported here. Female PD patients exhibited significantly higher antibody titers against both monomeric and aggregated α-synuclein compared with male patients, indicating a more robust humoral immune response in females (Figure 8).

### 3.3. Serum Metabolomic Profiling

To investigate systemic metabolic alterations associated with PD and their modulation by biological sex, ^1^H NMR-based metabolomic profiling was performed on serum samples.

**Figure 9:**
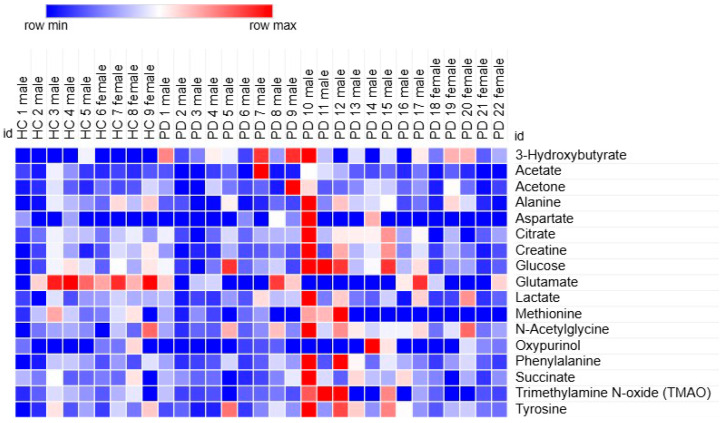
Heatmap of the most relevant metabolites in healthy controls (HC) and Parkinson’s disease (PD) patients, stratified by sex. Data are row-normalized (minimum = 0, maximum = 1). Red indicates higher relative abundance, whereas blue indicates lower relative abundance. The metabolites shown were selected based on statistical significance. *p* < 0.05.

An exploratory analysis of spectra was first made using principal component analysis (PCA) to reveal the main trends in our ^1^H NMR data set. No clear tendency of the samples to separate according to HC and PD groups was observed (Supplementary Figure S4A). The results of the discrimination were strengthened by PLS-DA, where the HC group was discriminated from the PD group by a two-component model with an R^2^X (cum) of 0.642, an R^2^Y (cum) of 0.631 and a Q2 of 0.414 (Supplementary Figure S4B). The analysis of the discriminant metabolites obtained by the application of PLS-DA allowed the identification of a total of 17 metabolites that were found to be significantly altered between PD patients and HC, highlighting a substantial metabolic remodeling associated with the disease (Figure 9).

**Figure 10:**
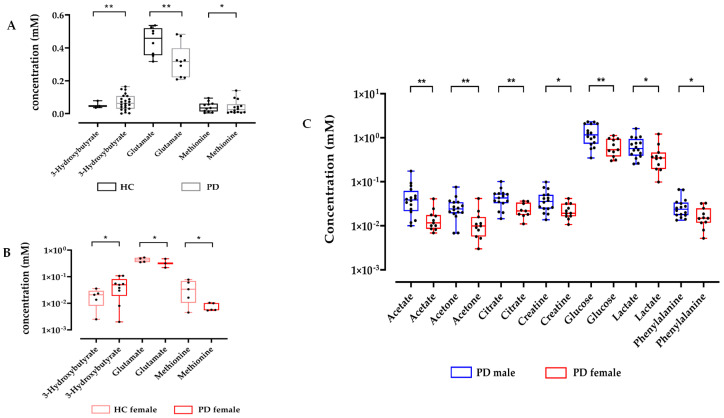
Evaluation of significantly altered metabolites in serum samples from healthy controls (HC) and Parkinson’s disease (PD) patients. (**A**) Comparison between HC and PD subjects regardless of sex. (**B**) Comparison between HC and PD female subjects. (**C**) Comparison between PD male and PD female subjects. Metabolite concentrations were determined by ^1^H-NMR spectroscopy. Data are presented as box-and-whisker plots showing median, interquartile range, minimum and maximum values, together with individual data points. Statistical analysis was performed using the Wilcoxon-Mann-Whitney test. Significant differences are indicated as * *p* < 0.05 and ** *p* < 0.01.

When analyzed independently of sex, PD patients exhibited increased levels of 3-hydroxybutyrate (BHB) and reduced concentrations of glutamate and methionine compared with HC (Figure 10A). Female PD patients showed a significant increase in BHB levels compared with female HC, indicating enhanced ketone body production (Figure 10B). No significant differences were detected between PD and HC males (Supplementary Figure S5). Male PD patients showed higher concentrations of acetate, acetone, citrate, creatine, glucose, lactate, and phenylalanine compared with female PD patients, indicating broader dysregulation of energy and amino acid metabolism (Figure 10C).

## 4. Discussion

The identification of reliable, minimally invasive biomarkers remains a major challenge in Parkinson’s disease research. In this context, the development of circulating biomarkers is crucial not only to support clinical diagnosis but also, if validated, to play a key role in guiding therapy in addition to enabling patient stratification and the early detection of disease subtypes characterized by distinct molecular mechanisms [1,2,4].

In this pilot study, we employed an integrated biochemical and metabolomic approach on serum samples from HC and PD patients to investigate whether systemic molecular alterations in PD patients differ between males and females. Our findings provide preliminary evidence of sex-related differences across multiple molecular domains, including oxidative stress, inflammatory signaling, α-synuclein-related measures, immune responses, and metabolic pathways.

Oxidative stress, driven by mitochondrial dysfunction and impaired protein clearance, is a key mechanism in neurodegeneration [24]. Although no significant differences in systemic oxidative stress markers were observed between PD patients and HC, specific components of the antioxidant defense system appear to be altered. Notably, reduced Nrf2 expression in female PD patients suggests a potential sex-dependent modulation of antioxidant responses, which may be compatible with altered antioxidant responses; however, the functional implications remain unclear [25]. Consistently, the physiological sex-related difference in SOD2 expression observed in controls was lost in PD patients, indicating a disruption of baseline mitochondrial antioxidant dimorphism. While NQO1 levels did not differ significantly, their downward trend in PD patients, together with unchanged UCP2 expression, might point to a limited or dysregulated activation of downstream Nrf2-dependent pathways [26].

The inflammatory profile is consistent with the presence of sex-related differences in systemic inflammatory markers in PD. Serum IL-6 levels were significantly elevated in PD patients, with a more pronounced increase observed in males. This finding may indicate a greater inflammatory burden in male PD patients and is in line with previous evidence implicating age-related inflammatory processes in neurodegenerative diseases [27]. However, because IL-6 is a pleiotropic inflammatory cytokine and is not specific to PD, the contribution of factors other than PD cannot be completely excluded. Therefore, these observations should be interpreted with caution and warrant further investigation in larger and more comprehensively characterized cohorts.

Conversely, while MMP9 activity did not differ between groups, MMP3 levels were significantly higher in female PD patients compared with males, pointing to a possible sex-specific involvement of proteolytic pathways associated with extracellular matrix remodeling and neuroinflammatory processes. Notably, MMP3 has been implicated in α-synuclein processing and dopaminergic neurotoxicity [28,29].

The analysis of protease-resistant α-synuclein species and the corresponding humoral immune response provides additional insight into systemic features associated with PD. The detection of multiple proteinase K-resistant α-synuclein species in PD sera suggests that structurally distinct α-synuclein forms can be detected at the peripheral level, in agreement with previous reports highlighting α-synuclein heterogeneity and its potential biomarker value [30]. Among these, the ~52 kDa band, which was increased in male PD patients, may correspond to higher-order or modified α-synuclein assemblies with enhanced structural stability. However, the molecular nature and biological significance of this species remain to be established. Female PD patients, in contrast, exhibited higher antibody titers against both monomeric and aggregated α-synuclein species. These findings may indicate differences in the humoral response to α-synuclein between male and female patients [31], although the underlying mechanisms and their potential relevance to disease pathophysiology require further investigation. Given the known sensitivity of circulating α-synuclein measurements to pre-analytical variables, these observations should be interpreted cautiously.

The metabolomic analysis further supports the presence of systemic remodeling, consistent with previous metabolomic studies [32,33].

Female PD patients, compared to HC females, exhibited increased levels of 3-hydroxybutyrate (BHB) and reduced concentrations of glutamate and methionine, suggesting a shift in energy metabolism and a potential perturbation of amino acid-related redox pathways. The increased BHB levels may indicate enhanced ketone body utilization as a potential adaptive response to metabolic or oxidative stress. This observation appears consistent with the alterations in Nrf2 signaling and the enhanced humoral immune response observed in females, possibly reflecting the engagement of compensatory mechanisms.

Male PD patients, compared to female PD patients, exhibited broader metabolic changes, including increased levels of glucose, lactate, citrate, acetate, acetone, creatine, and phenylalanine, suggesting alterations in glycolysis, mitochondrial function, and amino acid metabolism. Altered phenylalanine metabolism has also been associated with PD in recent studies [34]. The coexistence of these metabolic alterations with changes in inflammatory markers and α-synuclein-related measures raises the possibility that these processes may be linked in PD [35].

This study has some limitations that should be acknowledged. First, the relatively small sample size, particularly after stratification by sex, may limit the statistical power and reduce the generalizability of the results. Therefore, the present work should be considered a pilot study and the reported differences should be interpreted as exploratory and hypothesis-generating. In addition, potential confounding factors, including age, hormonal status, and other clinical variables that may influence inflammatory, oxidative, and metabolic markers, cannot be completely excluded. Furthermore, given the number of biomarkers and metabolites investigated, some of the observed associations will require confirmation in independent cohorts. Despite these limitations, the convergence of observations obtained through multiple complementary analytical approaches suggests the biological relevance of the identified trends.

## 5. Conclusions

This pilot study suggests that biological sex may contribute to the systemic molecular heterogeneity observed in Parkinson’s disease. Our integrated analysis identified sex-related differences in inflammatory, oxidative, and metabolic profiles, with trends indicating a more pronounced inflammatory and metabolic dysregulation in males and distinct antioxidant and immune-related responses in females. Given the limited sample size and the exploratory nature of the study, these findings should be interpreted with caution and considered hypothesis-generating. Nevertheless, the results support the need for further investigations in larger, well-characterized cohorts to clarify the contribution of sex-specific molecular mechanisms to Parkinson’s disease pathophysiology and their potential relevance to precision medicine approaches.

## Data Availability

The data presented in this study are available on request from the corresponding author.

## Supplementary Materials

The following supporting information can be downloaded at https://www.mdpi.com/article/10.3390/biomedicines14071511/s1, Figure S1. Permutation plot of the PLS-DA model comparing healthy controls and Parkinson’s disease patients. Figure S2. Comprehensive densitometric analysis of α-synuclein serum profiles. Figure S3. Comprehensive evaluation of serum α-synuclein antibody levels. Figure S4. Multivariate metabolomic profiling of Parkinson’s disease and control sera by ^1^H NMR. Figure S5. Quantitative serum metabolomics profile of HC and PD patients.

## Abbreviations

The following abbreviations are used in this manuscript:

BAP	Biological Antioxidant Potential
BHB	β-Hydroxybutyrate
D-ROMs	Derivate-Reactive Oxygen Metabolites
DTT	Dithiothreitol
ELISA	Enzyme-Linked Immunosorbent Assay
HC	Healthy Controls
IL-6	Interleukin-6
MMPs	Matrix Metalloproteinases
MMP3	Matrix Metalloproteinase-3
MMP9	Matrix Metalloproteinase-9
MPD	2-Methyl-2,4-pentanediol
NMR	Nuclear Magnetic Resonance
NQO1	NAD(P)H Quinone Oxidoreductase 1
Nrf2	Nuclear Factor Erythroid 2–Related Factor 2
OD	Optical Density
PBS	Phosphate-Buffered Saline
PBST	Phosphate-Buffered Saline with Tween-20
PCA	Principal Component Analysis
PD	Parkinson’s Disease
PLS-DA	Partial Least Squares Discriminant Analysis
ROS	Reactive Oxygen Species
SD	Standard Deviation
SOD2	Superoxide Dismutase 2
TCA	Tricarboxylic Acid Cycle
TMB	3,3′,5,5′-Tetramethylbenzidine
TSP 3	3-(Trimethylsilyl) propionic Acid-d4 Sodium Salt
UCP2	Uncoupling Protein 2
