# Supplementary material for: Sex-Specific Systemic Signatures in Parkinson’s Disease: Integrated Biochemical and Metabolomic Evidence
Source: Biomedicines. 2026 Jul 4;14(7):1511. doi: 10.3390/biomedicines14071511 (PMC13404979; doi:10.3390/biomedicines14071511)
Supplement: Supplementary file 1 [file biomedicines-14-01511-s001.zip › biomedicines-4373953-supplementary.pdf]

biomedicines

MDPI

Supplementary Materials

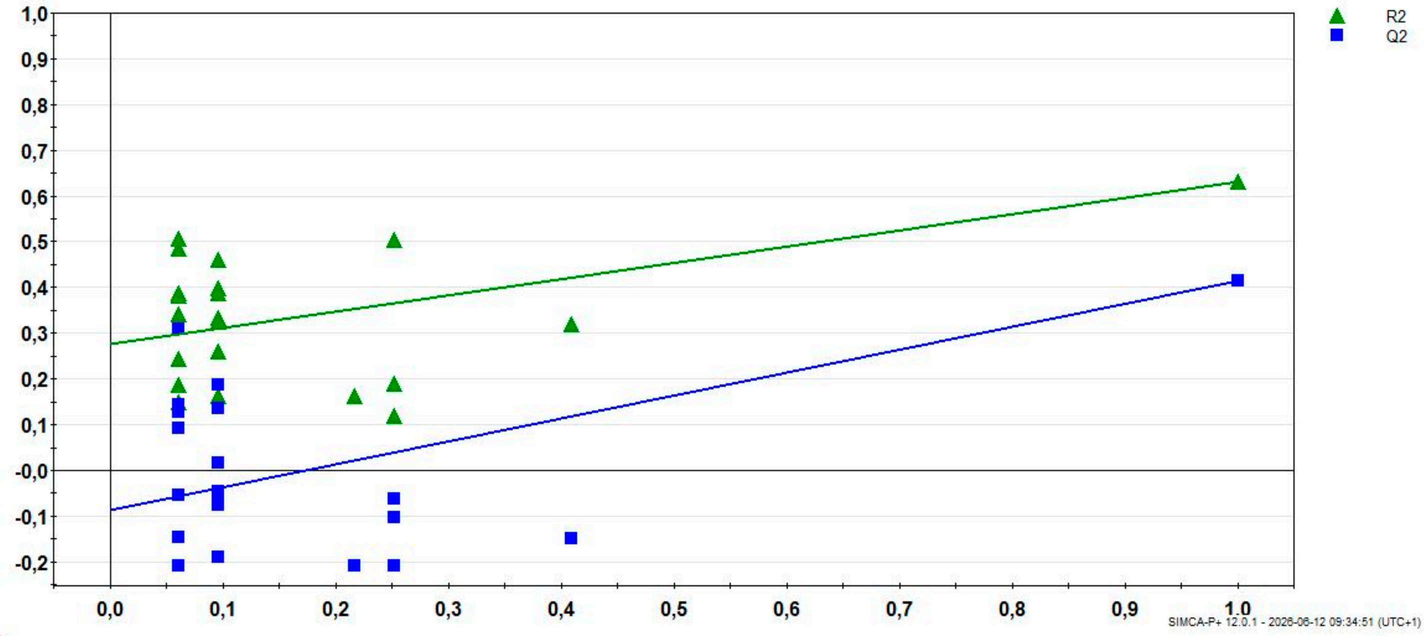

Figure S1. Permutation plot of the PLS-DA model comparing healthy controls and Parkinson's disease patients. Model validation was performed using permutation testing (20 permutations) to assess the risk of overfitting. The permutation analysis generated models with intercepts: R^2 = (0.0, 0.276) and Q^2 = (0.0, −0.088).

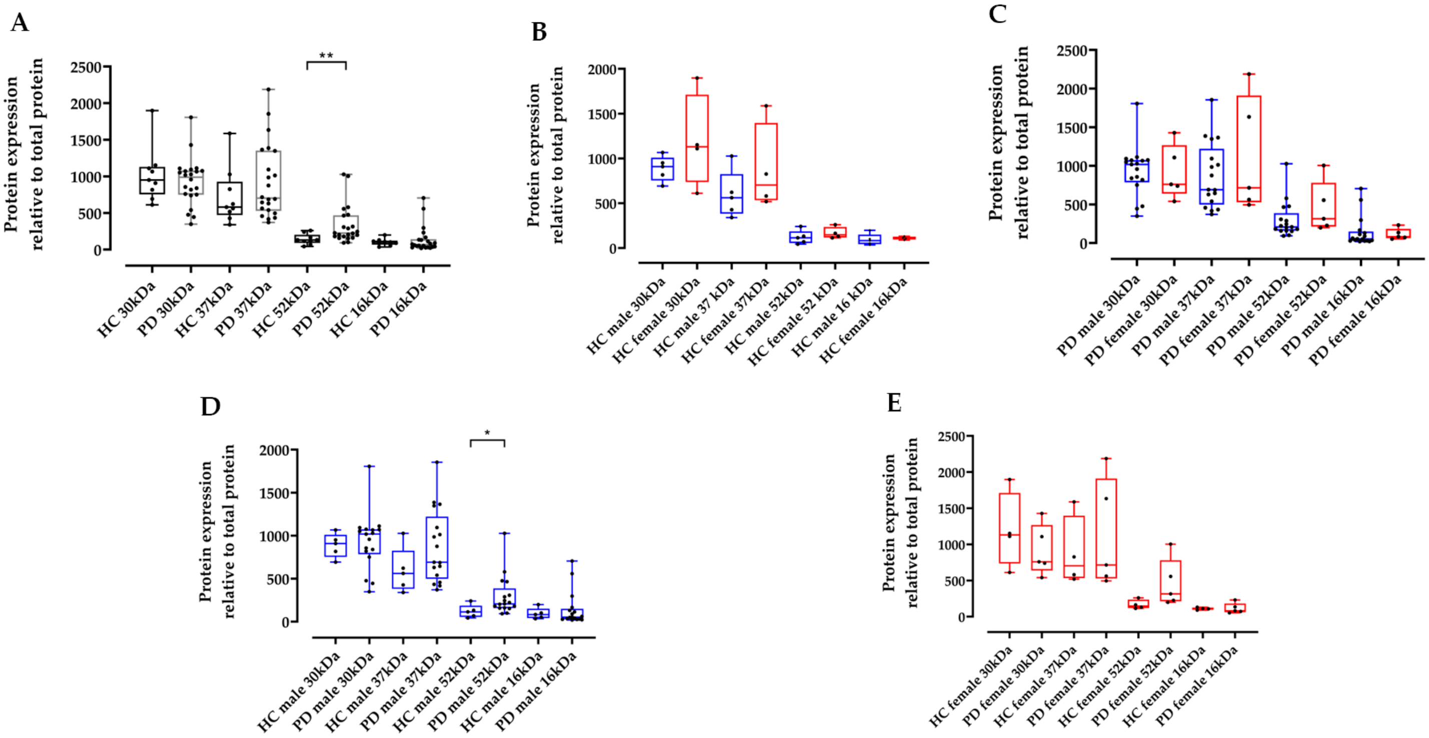

Figure S2 Comprehensive densitometric analysis of α-synuclein serum profiles. Densitometric quantification of all immunoreactive bands across the full molecular weight range in HC and PD patients following controlled proteinase K digestion. Data are presented for the whole cohort and stratified by sex (male and female). Protein levels were normalized to total protein loading. Data are presented as box-and-whisker plots showing median, interquartile range, minimum and maximum values, together with individual data points. Statistical analysis was performed using the Wilcoxon–Mann–Whitney test. Consistent with the main findings, no additional statistically significant differences were observed beyond the 52 kDa species reported in the main text (*$p < 0.05$; **$p < 0.01$).

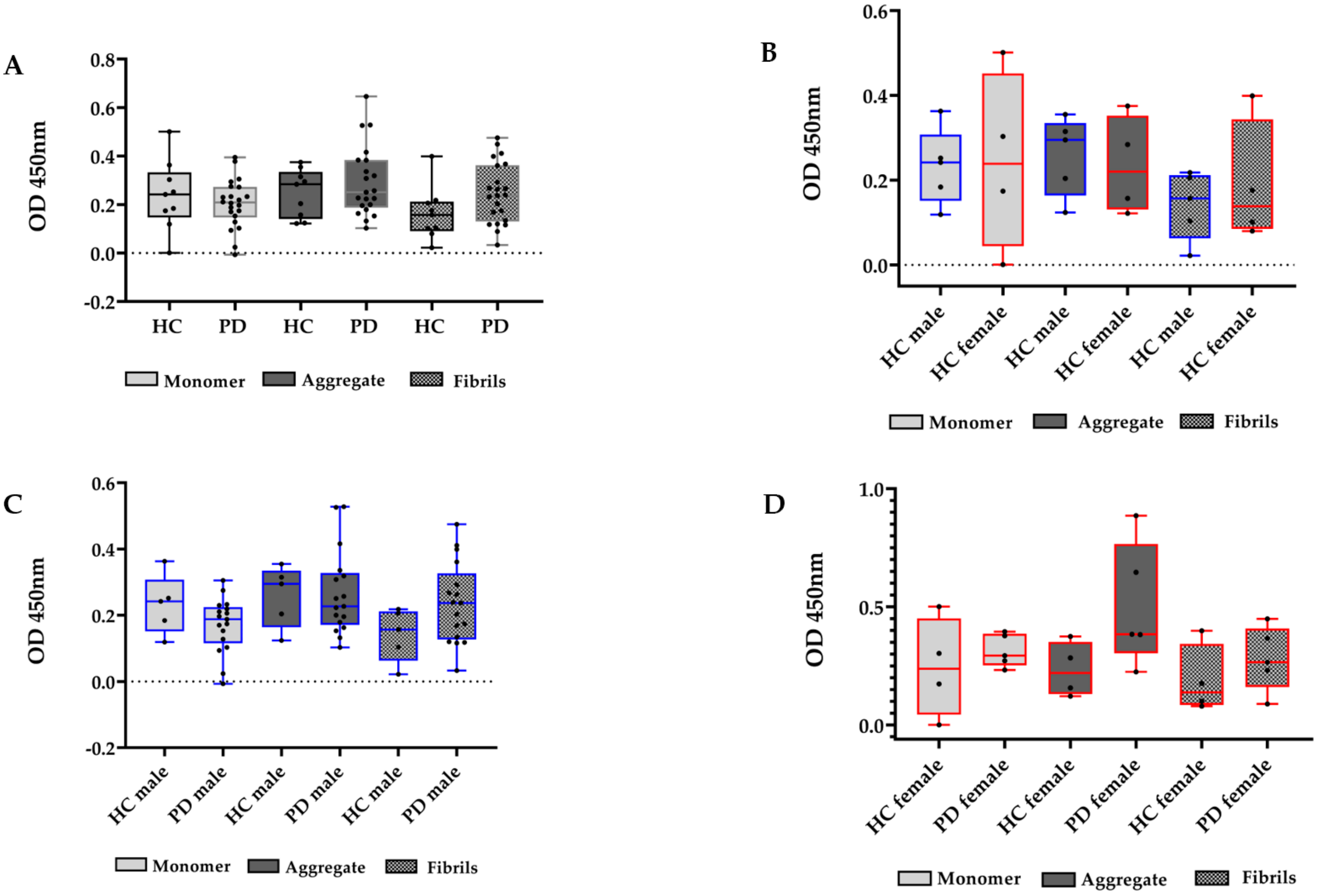

Figure S3. Comprehensive evaluation of serum α-synuclein antibody levels. α-Synuclein antibodies in serum samples from healthy controls (HC) and Parkinson's disease (PD) patients against different forms of α-synuclein was measured by an in-house indirect ELISA. Data are shown for comparisons between HC and PD subjects, both regardless of sex and stratified by sex (male and female). Data are presented as box-and-whisker plots showing median, interquartile range, minimum and maximum values, together with individual data points. Statistical analysis was performed using the Wilcoxon–Mann–Whitney test. No additional statistically significant differences were observed beyond those reported for PD male and female in the main text (*$p < 0.05$; **$p < 0.01$).

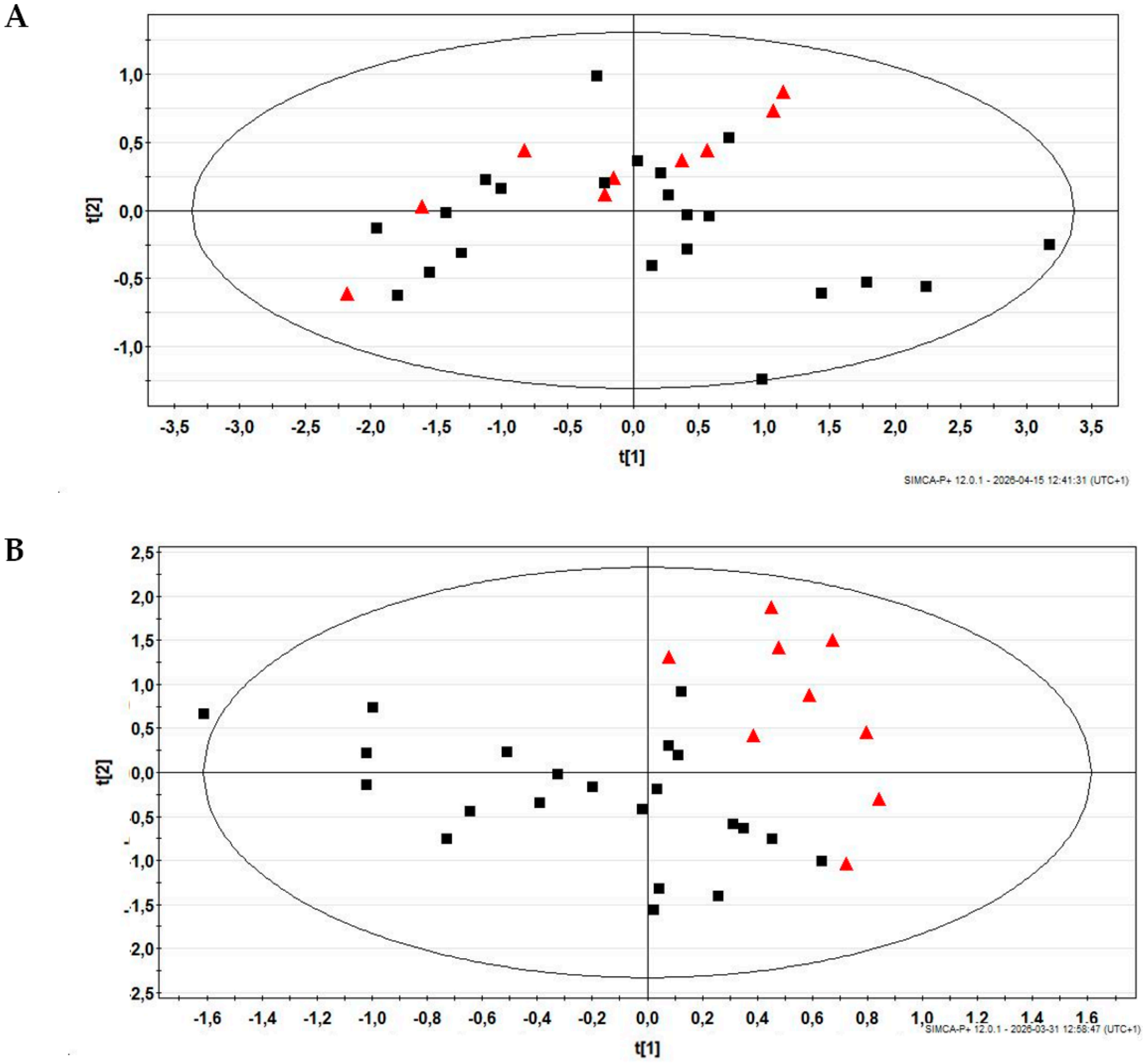

Figure S4. Multivariate metabolomic profiling of Parkinson's disease and control sera by ^{1}H NMR. (A) Principal component analysis (PCA) score plot obtained from ^{1}H NMR data. PCA resulted in a two-component model with an R2X value of 0.656, and a Q2 of 0.407. (B) Partial least squares discriminant analysis (PLS-DA) scores plot from ^{1}H NMR data. PLS-DA resulted in a two-component model with an R2X (cum) of 0.642, an R2Y (cum) of 0.631 and a Q2 of 0.414. Healthy control, red triangles, n=9; Parkinson disease, black boxes, n=22.

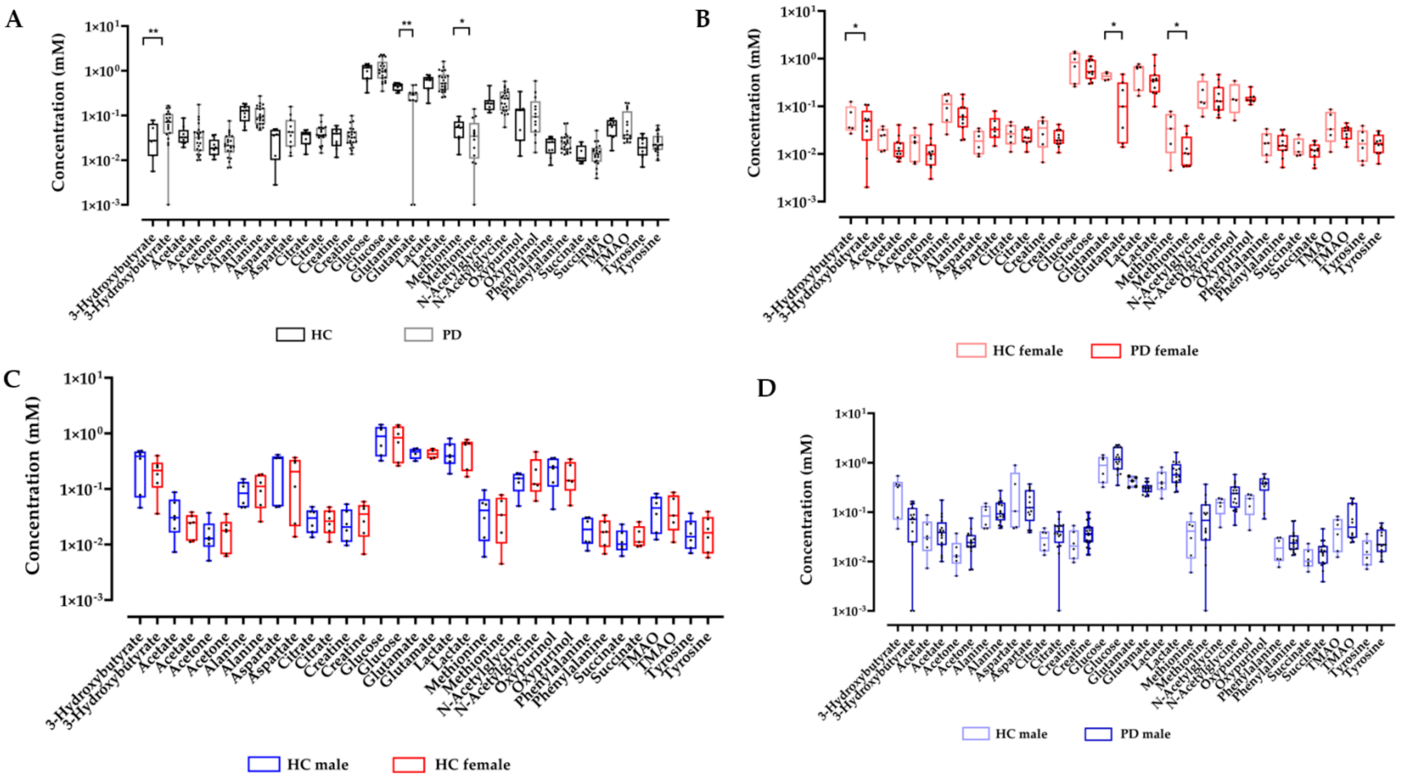

Figure S5. Quantitative serum metabolomics profile of HC and PD patients. Histograms represent the concentrations (mM) of metabolites in healthy controls (HC) and Parkinson's disease (PD) patients, analyzed both as a whole cohort and stratified by sex. Data were obtained via 1H-NMR spectroscopy. Data are presented as box-and-whisker plots showing median, interquartile range, minimum and maximum values, together with individual data points. Statistical significance was assessed using the Wilcoxon–Mann–Whitney test (*p < 0.05; **p < 0.01).
